# Examining oral pre-exposure prophylaxis (PrEP) literacy among participants in an HIV vaccine trial preparedness cohort study

**DOI:** 10.1186/s12913-022-08730-8

**Published:** 2022-11-10

**Authors:** Rujeko Samanthia Chimukuche, Rachel Kawuma, Nteboheleng Mahapa, Smanga Mkhwanazi, Nishanta Singh, Samantha Siva, Eugene Ruzagira, Janet Seeley, On behalf of the PrEPVacc Study Group, Glenda Gray, Nishanta Singh, Zakir Gaffoor, Neetha Morar, Thandiwe Sithole, Kubashni Woeber, Samantha Siva, Eldinah Hwengwere, Rujeko Samanthia Chidawanyika, Nteboheleng Mahapa, Phindile Khanyile, Ilesh Jani, Edna Viegas, Isabel Remane, Odete Bule, Edna Nhacule, Patricia Ramgi, Raquel Chissumba, Eduardo Namalango, Yolanda Manganhe, Carmelia Massingue, Igor Capitine, Jorge Ribeiro, Lucas Maganga, Wiston William, Emmanuel Kapesa, Elizabeth Danstan, Doreen Pamba, Marco Missanga Amani Kway, Abisai Kisinda, Lilian Njovu, Lwitiho Sudi, Revocatus Kunambi, Said Aboud, Patricia Munseri, Eligius Lyamuya, Frank Msafiri, Agricola Joachim, Edith Tarimo, Diana Faini Tumaini Nagu, Deus Buma, Muhammad Bakari, Pontiano Kaleebu, Freddie Mukasa Kibengo, Ayoub Kakande, Jennifer Serwanga, Rachel Kawuma, Christian Hansen Holmes, Sheila Kansiime, Eugene Ruzagira, Janet Seeley, Sylvia Kusemererwa, Sylvia Masawi, Vincent Basajja, Tobias Vudriko, Peter Hughes, Shamim Nabukenya, Gertrude Mutonyi, Rita Nakiboneka, Susan Mugaba, Jonathan Weber, Cherry Kingsley, Tom Miller, Sheena McCormack, Angela Crook, David Dunn, Henry Bern, Aminata Sy, Liz Brodnicki, Sarah Joseph, Claire Wenden, Kundai Chinyenze, Jacqueline Musau, Mabela Matsoso, Mary Amondi, Paramesh Chetty, Anne Gumbe, Giuseppe Pantaleo, Song Ding, Charlotta Nilsson, Arne Kroidl, Julie Fox, Gustavo Doncel, Allison Matthews, Jim Rooney, Carter Lee, Merlin Robb

**Affiliations:** 1 Africa Health Research Institute, KwaZulu-Natal, South Africa; 2 Social Aspects of Health Programme, MRC/UVRI and LSHTM Uganda Research Unit, Entebbe, Uganda; 3 Gender and Health Research Unit, South African Medical Research Council, Durban, South Africa; 4 HIV and Other Infectious Diseases Research Unit, South African Medical Research Council, Durban, South Africa; 5 HIV Epidemiology and Interventions Programme, MRC/UVRI and LSHTM Uganda Research Unit, Entebbe, Uganda; 6 Faculty of Epidemiology and Population Health, London School of Hygiene and Tropical Medicine, London, UK; 7 Department of Global Health and Development, London School of Hygiene and Tropical Medicine, London, UK

**Keywords:** HIV, Pre-exposure prophylaxis, PrEP literacy

## Abstract

**Background:**

PrEP literacy is influenced by many factors including the types of information available and how it is interpreted. The level of PrEP literacy may influence acceptability and uptake.

**Methods:**

We conducted 25 in-depth interviews in a HIV vaccine trial preparedness cohort study. We explored what participants knew about PrEP, sources of PrEP knowledge and how much they know about PrEP. We used the framework approach to generate themes for analysis guided by the Social Ecological Model and examined levels of PrEP literacy using the individual and interpersonal constructs of the SEM.

**Results:**

We found that PrEP awareness is strongly influenced by external factors such as social media and how much participants know about HIV treatment and prevention in the local community. However, while participants highlighted the importance of the internet/social media as a source of information about PrEP they talked of low PrEP literacy in their communities. Participants indicated that their own knowledge came as a result of joining the HIV vaccine trial preparedness study. However, some expressed doubts about the effectiveness of the drug and worried about side effects. Participants commented that at the community level PrEP was associated with being sexually active, because it was used to prevent the sexual transmission of HIV. As a result, some participants commented that one could feel judged by the health workers for asking for PrEP at health facilities in the community.

**Conclusion:**

The information collected in this study provided an understanding of the different layers of influence around individuals that are important to address to improve PrEP acceptability and uptake. Our findings can inform strategies to address the barriers to PrEP uptake, particularly at structural and community levels.

**Trial registration:**

https://clinicaltrials.gov/ct2/show/NCT04066881

**Supplementary Information:**

The online version contains supplementary material available at 10.1186/s12913-022-08730-8.

## Introduction

In 2022 HIV prevalence in South Africa is approximately 13.9%, with the total number of people living with HIV (PLWHIV) estimated to be approximately 8,45 million [1]. South Africa became the first country in Africa to register and provide oral pre-exposure prophylaxis (PrEP) for HIV prevention in 2016 [2], beginning with the provision of PrEP to sex workers [3]. The 2017-2022 National Strategic Plan proposes to expand PrEP distribution to other people who are at high risk of HIV infection such as men who have sex with men (MSM) and adolescent girls and young women [4, 5]. In the course of this roll-out a number of studies have been conducted on the attitudes to, knowledge, acceptability and use of PrEP among young people, women, and girls in South Africa [6–11]. Globally, almost a million people initiated oral PrEP by the end of 2020, with over 100,000 people in South Africa [12].

PrEP effectiveness largely depends on having accurate, up to date information about the forms of PrEP and its availability and PrEP adherence [13]. Whilst individuals may be aware of PrEP, they may understand and interpret its use differently depending on the information that is provided or conveyed to them as well as their own personal interpretation [14]. Research on factors influencing PrEP uptake reveal that knowledge does not necessarily translate into acceptance and behaviour change [2, 15–17].

To increase uptake of PrEP in South Africa, efforts have been made to raise awareness and increase PrEP literacy by distributing information nationally [5]. For example, the National Department of Health has used a phased approach to distribute information through communication programmes in schools, health facilities, workplaces, and community centres [5]. Information, Education and Communication (IEC) materials with information about PrEP are distributed via interpersonal communication, mass and social media communications by ward-based outreach teams and community-based organisations facilitating a knowledge sharing platform for educating and communicating between health providers and patients [5]. Despite these efforts, and the availability of PrEP at clinical research centres, health facilities and pharmacies, knowledge and community awareness of PrEP in South Africa is still limited [2, 9, 18, 19].

Health literacy is the extent to which individuals have the capacity to acquire, process, and understand information and services available and how this influences their health decisions and uptake [20]. When assessing whether an individual will take up PrEP, it is important to not only consider how much information they have attained but also whether that knowledge influences their decision to either use or not use the drug. We recognise, however, that PrEP knowledge is one factor amongst other indicators of PrEP use, and that increasing functional knowledge about PrEP may not be sufficient for increasing their decision to use PrEP [21]. For example, DiTullio and colleagues showed that providing information about PrEP did not translate into increased use among MSM, and there was a need to devise more end user focused approaches for PrEP promotion [22].

In this study, we assessed the concept of ‘PrEP literacy’ through gathering data on the knowledge accessed, the use made of PrEP and people’s perceptions of PrEP, which may have an impact on uptake.

**Fig. 1:**
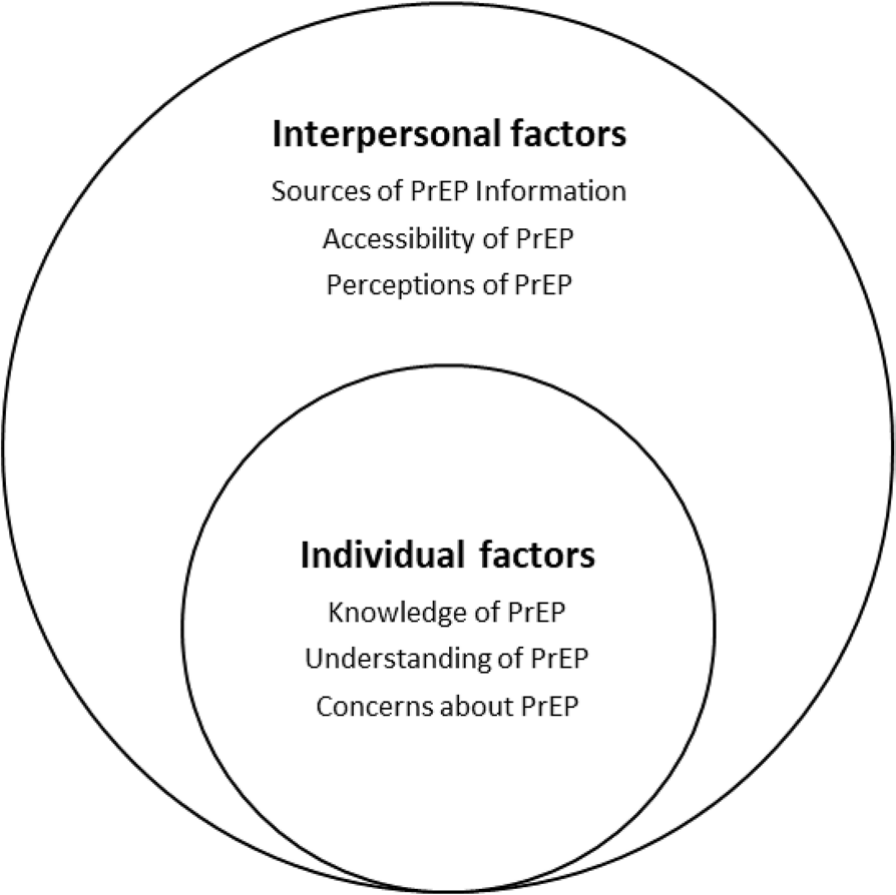
Modified Socio-Ecological Model levels of influence on PrEP literacy

The Socio-Ecological Model (SEM) provides a framework to consider the complex interplay across individual, community, societal and structural factors which influence people’s behaviour. Using the SEM we focus on health literacy beyond the individual, looking at the delivery of health information, the type of knowledge provided to the public, the communication skills of healthcare professionals, and the health policies that surround an individual [23, 24]. We have used a modified version of the SEM in this study to analyse data from participants in the  HIV vaccine trial preparedness cohort. The cohort was established in preparation for the PrEPVacc trial, a phase IIb three-arm, two-stage HIV prophylactic vaccine trial with a second randomisation to compare Tenofovir alafenamide (TAF) plus emtricitabine (FTC) (TAF/FTC) to Tenofovir desoproxil fumarate/emtricitabine (TDF/FTC) as PrEP [25]. The SEM provides a framework to illustrate how PrEP use behaviour is influenced by different social systems on multiple levels [21]. We adapted the SEM to explain that while information is available on PrEP, individual and interpersonal factors play a part in explaining the level of understanding of PrEP and the PrEP uptake among cohort participants (Fig. 1).

Using this modified SEM, and focusing on individual and interpersonal levels, we were able to understand the different factors that influence knowledge and perceptions of PrEP and the impact on PrEP literacy. It was an important part of the preparation for the HIV vaccine trial to determine the uptake and adherence to PrEP to inform the development of tools to support adherence. During the vaccine trial study participants can access PrEP during the first 40 weeks of the trial to support the immunisation period.

## Methods

### Study design and setting

This qualitative methods study was nested in an ongoing prospective HIV vaccine trial preparedness study in Durban, South Africa. Individuals who were HIV negative (18 to 45 years) at high risk of HIV acquisition were recruited into the cohort between 2019 and 2021. Cohort participants were recruited from two areas in Durban known to have a high burden of HIV, referred to here as ‘Site A’ and ‘Site B’. HIV risk was measured at baseline using a questionnaire that included items on the number of sexual partners, condom use, use of alcohol and recreational drug use and history of sexually transmitted infections. Cohort participants are followed every three months for at least 12 months.

### Participants, sample selection and data collection

Participants were purposively selected on the basis of gender, age, location and occupation to take part in the in-depth interviews (IDIs). Data collection was conducted at cohort enrolment between July and October 2019 at Site A and between November 2020 and February 2021 (delayed by the COVID-19 pandemic) at site B. A semi-structured topic guide was used to encourage participants to respond to questions on broad areas of the study such as knowledge and perceptions of PrEP.

Two experienced social science researchers (initially both men, and then part way through the study a woman replaced one man) conducted the IDIs face to face at the clinical research site in IsiZulu (the main local language). Each interview lasted between 45-60 minutes. Participants were briefed on the purpose of the study and the research assistants checked on participants understanding of the study before seeking informed consent. Participants reviewed the participant information documentation prior to giving their written informed consent to be involved. All interviews were digitally recorded, transcribed and translated into English. Debriefing meetings were conducted after each interview between the interviewers and the lead author of this paper to improve probing, provide clarity on emerging themes and also refine the topic guide where necessary.

### Data analysis and interpretation

Data analysis was done manually, led by the first author (RSC), and supported by (RK), and the research assistants who collected the data. The coding framework was developed by the team based on four scripts which the entire team read and used to identify emerging codes. To increase inter-rater reliability and validity, the main codes were reviewed, discussed and agreed upon by consensus. For this paper the codes with similar meanings were merged to form the two major themes identified namely: knowledge about PrEP and perceptions of PrEP. Thereafter, for this particular analysis a matrix coding framework was developed in excel on to which data were manually coded by pasting illustrative quotes from the interviews against matching themes. All data were anonymised, and participants are identified only by their sex and age in this paper.

## Results

A total of 45 participants with ages ranging from 18 to 36 years were enrolled (site A, 20; site B, 25). Of these, 23 (51.1%) were female, 44 (97.7%) were single, 33 (73.3%) were unemployed and 21 (46.7%) had completed either their school leaving qualification or tertiary education. As noted above, two main themes provide the focus for the data used in this paper: knowledge of PrEP and perceptions of PrEP. We present the findings against each theme below using illustrative quotes.

### Knowledge about PrEP

#### Understanding of PrEP at the individual level

Individual level factors are biological characteristics that are associated with one’s own vulnerability [26]. These factors either positively or negatively influence the individual’s decision-making about taking PrEP.

In this study, at the individual level the participants indicated that they had knowledge of PrEP and understood that it should be taken to prevent sexual transmission of HIV but were not sure about its effectiveness. Information on PrEP was available, but participants had different understandings regarding its effectiveness as illustrated below:

*“It prevents you from having it, as they say, although I wouldn’t know if it is 100% working, or if it’s in testing or but I heard that, that there is a pill that helps you not to get infected”.* Male, 22 years old.

Participants indicated that they had little exposure to PrEP information prior to enrolment in the HIV vaccine trial preparedness study:

*“…The first time I heard about it was when I started the study with [xxx]. So no - there is no other place where I heard about it. So [the study] was my first time, which is last year”.* Male, 22 years old.

The information that participants gained about PrEP contributed to the opinion they formed around it. For instance, some commented that PrEP is meant for sexually active individuals who are at high risk of getting infected with HIV, as expressed below:

*“...in my knowledge ehh it is used by someone who knows that they are sexually active or is at high risk so that they do not get infected with STIs, such a person ehh would be able to get in from the clinic, they said it is available, they can get it from there so that they can use it”.* Female, 29 years old.

#### Concerns about PrEP

These opinions were formed by participants through discussions within their communities where they were able to express their fear of PrEP side effects based on the information they had gained during the study preparation. Participants mentioned persistent side effects as being among possible barriers to uptake of oral PrEP.

*“…Yah we also learned that like you don’t have to rely on them because they have side effects, I had personally posed a question, then they explained that if you are taking them for the first time they have side effects, others get semi-dizzy, others  say you shouldn’t take them, they make you nauseous you see”* Female, 27 years old.

#### Sources of PrEP information

Interpersonal factors are the person to person contacts in their social networks that individuals are exposed to around them. Interpersonal factors can provide social support and reinforce social norms and behaviour that serve as protective factors, although they can also have a negative influence, and a person may, as a result of the influence of others, stop doing something that is protective and desirable.

We found that participants talked of low PrEP literacy in their communities, which they said was attributed to limited exposure to information about PrEP and the lack of PrEP champions in the areas they stayed. In this way, participants highlighted that the community is deprived of the social networks, and interpersonal communication, that could provide information that would assist them in deciding about their own sexual health.

*“…I think it’s going to take a while, they will not just accept it [PrEP] If they don’t have lots of information about it.”* Male, 22 years old.

*“…Yah they don’t trust it [PrEP], they don’t know it because it has not been, ah-ah it is not yet popular/public, it has not been spoken about, there is no one, nothing like….No, the community, many people don’t know about PrEP, in my neighbourhood they don’t know about PrEP you see.”* Male, 26 years old.

Some participants highlighted the internet/social media as a source of knowledge about PrEP [27]. One participant mentioned the way in their community that social media was used for information to be circulated among friends:

*“…We read on social media and the internet as well we do get in, even friends we chat and say have you heard about something like this, yah. Yah something like that. Okay, so your friends are so informed? Yah they have knowledge”* Female, 29 years old.

#### Accessibility of PrEP

Structural factors, in particular, played a part in influencing the accessibility of PrEP at health facilities [26]; while the provision of PrEP may be among the services, it was not always necessarily straightforward to access. Some participants, for example, while acknowledging that PrEP is available at local health facilities, said that one had to request it from the health providers.

*“…you are the one who has to say that may I ask if PrEP is available its only then that they issue it, but it is available in my neighbourhood. It is at the clinic and research sites only where I know it is available, I have not heard of other places.”* Female, 27 *years*

However, participants also indicated that PrEP is sometimes out of stock, owing to a lack of demand, in health facilities and that prevented access.

*“…Yah most of the clinics some of them don’t have it to be honest. Even here at the local clinic sometimes when you get there they would tell you it is out of stock because people do not use it …”* Female 27 years

There was a view that some groups may face particular barriers accessing PrEP. Some participants expressed the view that the attitude of health care workers to young persons’ seeking PrEP, which may not be supportive, may negatively impact young people’s decision to take it.

*“…Ehhh it is not easy because they ask you questions as to why you need PrEP, where you learnt about it, how you know about it, how old you are, they don’t just take it out and give it to you”.* Female, 27 years old

As this quote, and the quote below indicate, some participants felt judged by the health workers when they ask about PrEP at their local health facilities.

*“...that at the clinic you get judged, you are so young like I mean 21 or 22, you already want to engage in sex without a condom or what do you want to do, you know.. so those are some of the things that prohibits us from going to the clinic...”* Male, 22 years old.

### Perceptions of PrEP

Stigma associated with HIV, and the association of pill taking with anti-retroviral therapy can limit the provision and/or uptake of HIV prevention, treatment, and care services. This stigma may exist at the interpersonal level, but also be pervasive at the wider societal level, or be perceived as being so by people fearing an association with HIV. Many participants highlighted the social stigma-related misperceptions regarding PrEP. An example is the perception that persons who take PrEP are living with HIV. This was a key barrier to uptake as participants feared this HIV related stigma.

*“…Ey, most of them are afraid that people will laugh at them. Ah, they are afraid that when people know that they are HIV positive, some of them could laugh at them and talk about them to other people. Yes, they are afraid of such things”* Male, 20 years old.

The environment in the community can either promote health and wellbeing or be a source of stigma in using and engaging in PrEP services. We found that misconceptions about PrEP were prevalent among participants’ friends and peers yet they were their primary source of knowledge and support. These views could potentially affect uptake and adherence to PrEP.

*“...people have a belief that says, why would they use pills when they do not have HIV....because other people sometimes think crazy things, they would say that the clinic wants to infect them with HIV because they want to kill us that is why they have brought us these PrEP pills..."* Male 20 years old*.*

## Discussion

Using the SEM we were able to highlight the influences of PrEP literacy and knowledge on PrEP uptake at individual and interpersonal levels . Our findings indicate that an understanding of the issues at the different levels of SEM regarding acceptability and uptake of PrEP can be used to guide the development of interventions.

Our setting was an HIV vaccine trial preparedness study and our findings provide insights into the PrEP literacy among the participants of that study, where the study team was their main source of PrEP information, although they were able to reflect on other possible sources of information in their social context. Our analysis has shown that PrEP literacy needs to be considered as more than simply the provision of information targeted at the individual level because of the influences in the wider environment that can affect uptake and adherence. Interpersonal and societal factors influence individuals’ knowledge and how they use that knowledge, affecting their health behaviours and choices in terms of PrEP acceptability and uptake. In this paper, we have shown that the availability of PrEP knowledge to study participants has not been enough to translate into uptake. Thus, an unwillingness to take PrEP, or to keep on taking PrEP, may not always be the result of a lack of knowledge; it is important to take account of the different influences on individuals.

Participants attributed low PrEP literacy in their communities (beyond the study context) to having limited access to information on PrEP within their communities. Similar findings have been reported by studies among MSM and other key populations [28–30]. Consistent with those previous studies, and as we note above, our participants reported they had little or no knowledge of PrEP prior to joining in the study [31]. Even so, the information provided in the study needed to be coupled with easy access to PrEP, and a supportive environment, where those accessing PrEP did not feel that their action was being judged.

Individual and interpersonal factors can be barriers to accessing healthcare services in general [17, 32, 33]. In the case of PrEP, available evidence shows that low PrEP literacy is caused by insufficient PrEP knowledge circulating in the communities [33]. However, access to knowledge alone does not affect uptake. Low uptake of PrEP can be influenced by individual and interpersonal factors including fear of side effects and stigma as outlined in the modified SEM model we used in our study.

Another study done in South Africa revealed that ensuring awareness of PrEP across multiple communication channels and promoting an interest in this form of HIV prevention increases uptake [9]. Other work has shown that disseminating health information through social networking sites can be highly effective since it generates public discussions among users and other community members that assist people in understanding and interpreting public health information [34]. Thus, there is need to broaden the platforms where health information is promoted with social media platforms, for example, being attractive channels to spread health information among young people [19]. Indeed, other work has shown that using social media facilitates rapid dissemination of health information over a wider community, at a low cost [21]. Although there are disadvantages, including the spreading of misinformation and a lack of acknowledgement of sources. However, given how widespread engagement with social media is among young people health care providers recognise the importance of social media websites and their potential in providing valuable health messages [21]. Finding ways to harness social media to counter misinformation is critical [35].

To assist in processing the available information provided, good patient–provider communication is an important factor contributing towards improving health and PrEP literacy [28]. Effective health communication between health providers and users encourages positive behaviour change and affects HIV prevention and treatment outcomes [36]. Health workers can be influential in the dissemination of PrEP information within communities and where PrEP is available at health facilities [37]; as our findings show, that influence can be supportive, but also detrimental to uptake where those distributing PrEP pass judgement, or are perceived to pass judgement, on those seeking PrEP. Health care workers may need intensified PrEP education, and instruction on self-management strategies to promote among PrEP users This can assist health workers to support users adequately and ensure high-uptake of PrEP within the general population [37].

Schools can also provide a starting place to conduct the PrEP educational/awareness programs targeting the adolescents and young people between ages of 15-24 who are at a high risk of HIV acquisition in South Africa [38]. School based education campaigns and community based sexual health programs can be used to spread PrEP education [34, 38]. Given the importance of clinics in providing PrEP and information about PrEP, strategies are needed to bridge the gap for young people between school-based knowledge on HIV prevention and accessing PrEP in health facilities. For the wider population facilitating easier access to PrEP as part of routine sexual and reproductive health care can support an increased understanding of its intended purpose and importance [22].

### Limitations of study

While a strength of this study was our ability to draw on data from a HIV vaccine trial preparedness cohort study, our limitation was that the interview topic guides were not originally tailored to focus on PrEP literacy and awareness. Therefore, we drew our findings form data provided on the broader prospective HIV vaccine trial preparedness cohort study. Another limitation is our inability to generalise to other populations since this was a clinical study setting where participants all received the same information on PrEP. The trial setting could have influenced our findings, notably social desirability of participants responses. 

### Recommendations

Based on our findings, we recommend that health care workers should be trained in different ways in which information on PrEP can be shared in the community. Information leaflets detailing PrEP can be made available to the public in several forms such as paper copies, shared on digital platforms, as well as provided through local radio programmes to reach the less-literate. In addition, PrEP users, may be encouraged to tell others about their experience and contribute to addressing HIV-related stigma in their communities.

## Conclusion

Using the SEM, we have highlighted different layers of influence that affect PrEP literacy, knowledge and acceptance. The perceived barriers to uptake greatly affect PrEP understanding and acceptability. It is important to increase the sources of reliable information to enhance PrEP knowledge. This can be done whilst addressing the barriers, particularly rumours and stigma, which affect uptake in communities. Gaining a better understanding of different influences is integral to understanding how to maximize the value of PrEP as an effective HIV prevention intervention in the general population, outside of the context of clinical trials.

## Supplementary Information

**Additional file 1.**

## Data Availability

The datasets generated and/or analysed during the current study are not publicly available due to confidentiality assured to our participants by protecting their anonymity but data is available from the corresponding author on reasonable request.

## Abbreviations

IDI: In-depth interviews
IEC: Information, Education and Communication
MSM: Men who have sex with men
PLWHIV: People living with HIV
PrEP: Oral pre-exposure prophylaxis
SEM: Socio-Ecological Model
TAF/FTC: Tenofovir alafenamide (TAF) plus emtricitabine
TDF/FTC: Tenofovir desoproxil fumarate/emtricitabine
