# Supplementary material for: Examining oral pre-exposure prophylaxis (PrEP) literacy among participants in an HIV vaccine trial preparedness cohort study
Source: BMC Health Serv Res. 2022 Nov 10;22:1336. doi: 10.1186/s12913-022-08730-8 (PMC9649006; doi:10.1186/s12913-022-08730-8)
Supplement: Supplementary file 1 — Additional file 1. [file 12913_2022_8730_MOESM1_ESM.pdf]

Development of an HIV Negative Registration Cohort for Future Participation in an HIV Vaccine Study.

IDI INTERVIEW GUIDE

[EXPLAIN THE FOLLOWING]

- This will be a 45mins-1 hr activity.
- I would like to hear your views. There are no right or wrong answers.
- Please feel free to give your ideas
- Any question you feel uncomfortable about, please feel free not to answer it. However, for purposes of learning, we would like to hear everything you want to say. Therefore, if this is of no harm to you, try and tell us everything.
- Your names will be kept confidential – when we write up the discussion, we never use people's real names. Instead, we will allocate a number which we shall use to identify you.
- The information you share with me will be between the two of us (emphasise that even the clinic team will not know what participant tells you)
- **I will be happy to answer any questions that may come up**
- Please put your cell phone/s on silent if possible
- Is it okay with you that we start the discussion?

NOTE: RECORD THE DATE AND TIME ACTIVITY STARTS

Background Information

[Age, Religion, Education, Marital status, job/Employment]

Recent life history

1. Please tell me a bit about your recent life history.
    - Work experiences
    - Places lived
    - Relationship history (family, partners and work)

Knowledge of HIV.

2. a) What do you know about HIV? (Personal perceptions)
    - Tell me about the different causes of HIV you know.
    - What are the different HIV prevention measures you know? (*probe for how much they know about these measures, access and uptake)*

    b) What do you know about PrEP?

    - Where do people access PrEP?
    - What influences/prevents the uptake of PrEP?

(If never had about PrEP-explain briefly about PrEP)

- What do you think about PrEP?
- If you were to access PrEP, where would you wish to get it from?
- -What would make it easier or difficult for one to take PrEP?

HIV Risk

3. Which types of people are most at risk? Why or which factors do put them at risk?
    a. Do you consider yourself as being at risk? Why? (dig deeper on the different activities)
    b. Also if one does not feel at risk, find out why?
    c. Is the risk in anyway related to his socio-demographic characteristics?

Acceptability to Study Procedures

4. Tell me about the study procedures you go through.
    a. What do you like or dislike about these procedures?
    b. What do you think should be done to improve these procedures?
    c. What do you think influences participation/non-participation in research studies?
    d. Why do people drop out?

5. What is this study/research all about?
6. What is your view of an HIV/AIDS vaccine? (Is it something that will help control spread of the virus/ will be easily accepted/ what are your fears? /expectations? Is it something that can easily be introduced to members of your community? Why/why not? Do you think it is something you would consider participating in? Why/why not? (Please use these probes even with people who don't know about the study)
    a. Had you heard about vaccines before joining the PrEPVacc study? (Probe from what sources?)

    b. What are the reasons for participating / not participating in vaccine trials?
    c. If you were to pass on information about the study to a friend, what would you tell him/her? *(Remember here we are more interested on how messages around participating in an HIV vaccine can be disseminated to the community. Not necessarily research study info)*

(*If do not know about the study)*

Thank you for taking your time to respond to our questions.

Do you have any questions?

Do you have anything else you would wish to talk about?

End

Manuscript: Examining oral pre-exposure prophylaxis (PrEP) literacy among participants in an HIV vaccine trial preparedness cohort study.

Consolidated criteria for reporting qualitative studies (COREQ): 32-item checklist

Developed from:
Tong A, Sainsbury P, Craig J. Consolidated criteria for reporting qualitative research (COREQ): a 32-item checklist for interviews and focus groups. *International Journal for Quality in Health Care*. 2007. Volume 19, Number 6: pp. 349 – 357

**No. Item**	**Guide questions/description**	**Reported on Page #**
**Domain 1: Research team and reflexivity**		
Personal Characteristics		
1. Interviewer/facilitator	Which author/s conducted the interview or focus group?	Page 1, line 1, line 6, line 6
2. Credentials	What were the researcher's credentials? E.g. PhD, MD	Page 8, line 165
3. Occupation	What was their occupation at the time of the study?	Page 8, line 165
4. Gender	Was the researcher male or female?	Page 8, line165-166
5. Experience and training	What experience or training did the researcher have?	Page 8, line 165
Relationship with participants		
6. Relationship established	Was a relationship established prior to study commencement?	Page 8, line 167-170
7. Participant knowledge of the interviewer	What did the participants know about the researcher? e.g. personal goals, reasons for doing the research	Page 8, line 167-170
8. Interviewer characteristics	What characteristics were reported about the inter viewer/facilitator? e.g. Bias, assumptions, reasons and interests in the research topic	Page 17,line 382-384

**Domain 2: study design**		
Theoretical framework		

9. Methodological orientation and Theory	What methodological orientation was stated to underpin the study? e.g. grounded theory, discourse analysis, ethnography, phenomenology, content analysis	Page 6, line 117-140
Participant selection		
10. Sampling	How were participants selected? e.g. purposive, convenience, consecutive, snowball	Page 7, line 159-160
11. Method of approach	How were participants approached? e.g. face-to-face, telephone, mail, email	Page 8, line 166
12. Sample size	How many participants were in the study?	Page 9, line 188
13. Non-participation	How many people refused to participate or dropped out? Reasons?	**None**
Setting		
14. Setting of data collection	Where was the data collected? e.g. home, clinic, workplace	Page 8, line 166-167
15. Presence of non-participants	Was anyone else present besides the participants and researchers?	**No**
16. Description of sample	What are the important characteristics of the sample? e.g. demographic data, date	Page 9, line 188-191
Data collection		
17. Interview guide	Were questions, prompts, guides provided by the authors? Was it pilot tested?	Page 8/Line 167-168
18. Repeat interviews	Were repeat inter views carried out? If yes, how many?	No
19. Audio/visual recording	Did the research use audio or visual recording to collect the data?	Page 8/Line 170-171
20. Field notes	Were field notes made during and/or after the inter view or focus group?	Page 8/Line 171-174
21. Duration	What was the duration of the inter views or focus group?	Page 8/ Line 167-168
22. Data saturation	Was data saturation discussed?	No
23. Transcripts returned	Were transcripts returned to participants for comment and/or correction?	No
**Domain 3: analysis and findings**		
Data analysis		

24. Number of data coders	How many data coders coded the data?	Page 9/Line 177-178
25. Description of the coding tree	Did authors provide a description of the coding tree?	Page 8/ Line 178-181
26. Derivation of themes	Were themes identified in advance or derived from the data?	Page 9/Line 181-186
27. Software	What software, if applicable, was used to manage the data?	N/A
28. Participant checking	Did participants provide feedback on the findings?	N/A
Reporting		
29. Quotations presented	Were participant quotations presented to illustrate the themes/findings? Was each quotation identified? e.g. participant number	Yes
30. Data and findings consistent	Was there consistency between the data presented and the findings?	Page 9/Line 194-Page 14/Line 307
31. Clarity of major themes	Were major themes clearly presented in the findings?	Page 9 / Line 194, Page 10/ Line 215, Page 10/Line 226 Page 12/Line 256 Page12/Line 286
32. Clarity of minor themes	Is there a description of diverse cases or discussion of minor themes?	Page 9/Line 195-Page 13/Line 307
